# Comparing Gastroesophageal Reflux Disease (GERD) and Non-GERD Patients Based on Knowledge Level of Acute Myocardial Infarction Symptoms, Risk Factors and Immediate Action Taken in Eastern Province, Saudi Arabia

**DOI:** 10.7759/cureus.35309

**Published:** 2023-02-22

**Authors:** Abdullah H Bohamad, Hadeel H Buali, Jinan M Aljasem, Ali H Alhussain, Mohammed A Alamer, Eman Elsheikh

**Affiliations:** 1 Medicine, King Faisal University, Alahssa, SAU; 2 Internal Medicine, College of Medicine, King Faisal University, Alahssa, SAU; 3 Cardiology, College of Medicine, Tanta University Hospital, Tanta, EGY

**Keywords:** first-aid, chest pain, non-cardiac chest pain, chest discomfort, gerd, mi, ami, st elevation myocardial ischemia, myocardial ischemia and infarction, gastroesophageal reflux disorder (gerd)

## Abstract

Introduction: A major cause of death globally is cardiovascular disease (CVD). Chest pain, nausea, vomiting, and heartburn are common symptoms of acute myocardial infarction (AMI). Chest pain is also the main symptom of gastroesophageal reflux disease (GERD). Therefore, the differential diagnosis of chest pain can become more challenging when GERD and AMI coincide. This study evaluated and compared the knowledge of the signs, symptoms, and immediate action that must be taken regarding AMI among GERD and non-GERD patients.

Methodology: An observational cross-sectional study using an online questionnaire was created and published between October and November 2022 to collect data from Saudi males and females 18 or older willing to participate in the study. Participants who were not Saudi had declined to complete the survey or had not fully completed it was excluded. The questionnaire contained three sections; after collecting informed consent, it made inquiries regarding respondents’ GERD status, demographic information, and knowledge and attitudes regarding GERD.

Results: This study included 691 responses from 300 non-GERD participants and 391 GERD participants. The study showed a high level of awareness (75.5%) of GERD, with significant differences in the level of awareness according to marital status, education levels, and occupation status. There was no significant difference in the level of awareness according to gender and GERD diagnosis, where the p-value > 0.05. The most common source of information about AMI was the Internet, followed by health care professionals. The most commonly known symptoms of AMI were sudden pain or discomfort in the chest, followed by a sudden shortness of breath.

Additionally, there was no significant association between the diagnosis of GERD and known risk factors. The association between GERD and other diseases (chi-square = 46.94, p-value 0.01). Obesity and smoking were the two main risk factors for heart attacks.

Conclusion: This study demonstrated that there was no significant difference between GERD and non-GERD participants regarding the knowledge and awareness level of AMI. Moreover, it showed that there was a lack of general knowledge and awareness of AMI in Saudi Arabia. The authors recommend initiating more awareness programs in Saudi Arabia to inform people about AMI and cardiovascular disease. More research is required to determine whether other patients are aware of AMI.

## Introduction

Cardiovascular diseases (CVD) are one of the leading causes of mortality worldwide, with approximately 17.9 million deaths reported annually [1]. Saudi Arabia reported a rise in CVD rates in recent years, with an overall prevalence of 5.5% [2]. Additionally, acute coronary syndrome (ACS) death rates in Saudi Arabia were 4%, 5.8%, and 8.1% in-hospital, at one month, and at one year, respectively [3]. Gastroesophageal reflux disease (GERD) results from the regurgitation of gastric contents into the esophagus [4]. More than 40% of adults in the United States suffer from GERD each month [5]. In Saudi Arabia, the estimated range of GERD prevalence is between 23.47% and 45.4% [6]. The classic symptoms of acute myocardial infarction (AMI) include chest pain, nausea, vomiting, and heartburn. These symptoms are also associated with GERD [7]. Moreover, the coexistence of GERD and AMI at the same time can make the differential diagnosis of chest pain more complicated [8]. GERD shares similar risk factors with AMI that include obesity, diabetes, hypertension, and hyperlipidemia, along with behavioral risk factors, including smoking and alcohol use [9-12]. Recent studies in Saudi Arabia showed a suboptimal awareness level of coronary artery disease (CAD) risk factors [13,14]. Since GERD is the most common cause of non-cardiac chest pain, it is crucial to assess public knowledge about the differences between heart attack and heartburn. No such work appears to have been published regarding such awareness among GERD patients. This study assesses and compares the understanding of early signs, symptoms, and appropriate responses in cases of AMI among GERD and non-GERD patients in Saudi Arabia.

## Materials and methods

Study design and selection criteria

An observational cross-sectional study was carried out using an online questionnaire to obtain responses from male and female citizens of Saudi Arabia who are older than 18 years of age and willing to participate in the study. Non-Saudi citizen participants, people who declined participation, and people who did not complete the entire questionnaire were excluded.

Questionnaire design

The questionnaire was designed in Arabic, as it is the native language of Saudi Arabia, and distributed randomly via Google Forms using social media platforms such as WhatsApp, Twitter, and Telegram. The questionnaire consisted of three sections and was presumed to take approximately three minutes to be completed. The questionnaire was developed and published between October and November 2022 and generated 691 responses, with 391 suffering from GERD who were self-diagnosed based on their symptoms and 300 who have never experienced such symptoms. The first section began with gathering informed consent from participants, followed by a query regarding the respondent’s status with regard to GERD. The second section addressed demographic data, including age, gender, marital status, educational level, and occupational status of respondents. Finally, the third section contained questions regarding the knowledge and attitude of GERD respondents' knowledge of and attitude toward AMI among GERD and non-GERD patients in Saudi Arabia.

Ethical consideration and statistical analysis

This study was approved by the Ethics Committee of King Faisal University with an ethical approval code KFU-REC-2022-SEP-ETHICS172. Participants were given a statement guaranteeing that their confidential information and privacy would be protected before proceeding to the questionnaire. Completion and submission of the questionnaire were considered as consent for inclusion in the study. Data were extracted, reviewed, coded, and entered into IBM Statistical Package for the Social Sciences (SPSS) software, version 22 (SPSS, Inc., Chicago, IL).

## Results

The results showed a significant difference in awareness level according to marital status; the highest awareness level was among single people and the lowest awareness was among widowed people (F= 3.602, p-value = 0.013). By educational level, the highest level of awareness was among bachelors and the lowest awareness level was among people with diplomas (F= 8.328, p-value < 0.01). By occupational status, the highest awareness level was among students and the lowest was among housewives (F= 8.328, p-value < 0.01). There were no significant differences in awareness according to gender or GERD diagnosis (p-value > 0.05) (Table 1). There were 691 participants between the ages of 15 and 86 (mean = 31.7, standard deviation = 13.66). The majority (56.4%) were female, and 43.3% were male. Regarding marital status: the majority (55.9%) were single, 42.5% were married, 1% were divorced, and 0.6% were widowed. On an educational level, the majority (58.8%) had a bachelor’s degree, 21% had secondary education, 8.4% had a diploma, 5.2% had postgraduate education, 4.8% had primary education, and 1.9% had intermediate education. The majority (41.8%) of respondents were students, 29.2% were employees, 11.3% were retired, 9.7% were housewives, and 8% were looking for a job. The majority (53.5%) earned monthly incomes of less than 3,000 SR, 23.4% had incomes from 3,000 SR to 10,000 SR, 15.5% had incomes from 10,000 SR to 20,000 SR, and 7.5% had incomes of more than 20,000 SR (Table 2). Results show that 56.6% have GERD diagnoses and 43.4% do not. Among people with GERD diagnoses, 37.6% had no other diseases, 5.2% had diabetes, 4.1% had dyslipidemia, 3.9% had hypertension, 1.4% had heart diseases, 0.45 had suffered a stroke, and 3.9% had other diseases. 51.4% of this cohort were aware of heart attacks and 5.2% had not. 38.9% of the people knew someone who had experienced a heart attack before and 17.7% did not. 39.8% had received information related to heart attacks and 16.8% had not. 52% knew that sudden heart attack requires prompt treatment and 4.6% did not. 28.4% would call an ambulance if they witnessed heart attack signs/symptoms, 24.6% would take the patient to the hospital, 2.9% will call a doctor and 0.7% would contact the patient’s family. 47.95% knew the phone number to contact an ambulance and 8.7% did not. 44.4% had heard about the risk factors of heart attack and 2.2% had not. Among people without GERD/heartburn diagnoses, 37.9% did not suffer from any diseases, 1.4% had diabetes, 0.4% had dyslipidemia, 0.7% had hypertension, 0.9% had heart disease, none had suffered a stroke, and 14% had other diseases. 40.1% knew about heart attacks and 3.3 % did not. 25.3% knew someone who had experienced a heart attack and 18.1% did not. 29.1% had received information related to heart attacks and 14.3% had not. 39.8% knew sudden heart attacks require prompt treatment and 3.6% did not. 21.1% would call an ambulance if they witnessed heart attack signs/symptoms, 20.7% would the patient to the hospital, 9% would call a doctor, and 0.3% would contact the patient’s family. 34.7 knew the phone number to contact an ambulance and 8.7% did not. 32.4% had heard about the risk factors of heart attack and 11% had not (Table 3). Levels of awareness among respondents regarding heart attack information were high (mean: 75.5%). The most identified item was that sudden heart attacks require prompt treatment (91.8%), then hearing about heart attacks (91.5%), and the least-known item was what to do first when witnessing signs/symptoms of heart attack (49.5%) (Table 4). There was a significant association between GERD diagnoses and other diseases (chi-square = 46.94, p-value < 0.01). There were also significant associations between this diagnosis and knowing someone who had experienced a heart attack before (chi-square = 8.094, p-value = 0.004). However, there were no significant associations between this diagnosis and other factors (hearing about heart attacks, receiving information related to heart attacks, knowing the need for prompt treatment for sudden heart attacks, knowing the proper actions to take when witnessing signs/symptoms of heart attack, knowing the ambulance phone number). The p-value for these factors was > 0.05 (Table 5). The most popular source of information about heart attack was the Internet (28.1%), followed by health care professionals (21%), books (15.8%), TV (13.9%), media (13.6%), seminars (4.8%), and promotional leaflets (2.8%) (Table 6). The most identified risk factor for heart attack among respondents was obesity (14.5%), followed by smoking (13.9%), heart diseases (11.6%), high cholesterol (9.8%), stress (9.1%), diabetes (8.4%), unhealthy diet (8%), lack of exercise (7.2%), alcohol (6.1%), genetics (5.5%), atrial fibrillation (4.6%), and exercise (0.9%). 0.5% were not aware of any risk factors (Table 7). The most frequently identified symptoms of heart attack were sudden pain or discomfort in the chest (26.2%), sudden shortness of breath (21.2%), sudden pain or discomfort in the arms or shoulders (16.9%), sudden pain or discomfort in jaw, neck, or back (15.2%), weakness or dizziness (11.2%) and sudden disturbance of the vision in one or both eyes (9.2%) (Table 8). 

**Table 1:** Difference in awareness level

Variable	Test	Categories	Mean	Statistics	P-value
Gender	Independent Samples Test	Male	1.598	-1.018	0.309
		Female	1.615		
Marital Status	ANOVA	Single	1.6299	3.602	0.013
		Married	1.5821		
		Divorced	1.5306		
		Widowed	1.4643		
Educational Level	ANOVA	Primary	1.6364	4.216	0.001
		Intermediate education	1.4176		
		Diploma	1.532		
		Secondary	1.597		
		Bachelor	1.6256		
		Postgraduate	1.6111		
Occupational Status	ANOVA	Employee	1.5898	8.328	0.000
		Student	1.6466		
		Looking for job	1.5714		
		Retired	1.6337		
		Housewife	1.4925		
Monthly Income	ANOVA	Less than 3000 SR	1.6116	0.865 0.459	
		From 3000 SR – 10000 SR	1.5847		
		From 10000 SR – 20000 SR	1.6222		
		More than 20000 SR	1.6209		
Diagnosis with GERD	Independent Samples Test	Yes	1.6215	-1.916	0.56
		No	1.5895		

**Table 2:** Demographic data

Variable	Category	Frequency	Percent
Gender	Male	301	43.6
	Female	390	56.4
Marital Status	Single	386	55.9
	Married	294	42.5
	Divorced	7	1.0
	Widowed	4	0.6
Education Level	Primary	33	4.8
	Intermediate Education	13	1.9
	Diploma	58	8.4
	Secondary	145	21.0
	Bachelor	406	58.8
	Postgraduate	36	5.2
Occupational Status	Employee	202	29.2
	Student	289	41.8
	Looking for job	55	8.0
	Retired	78	11.3
	Housewife	67	9.7
Monthly Income	Less than 3,000 SR	370	53.5
	From 3,000 SR – 10,000 SR	162	23.4
	From 10,000 SR – 20,000 SR	107	15.5
	More than 20,000 SR	52	7.5

Age	Min	Max	Mean	Standard Deviation
	15	89	31.7	13.66

**Table 3:** Comparison among people who have/have not been diagnosed with GERD

Variables	Have you ever been diagnosed with GERD or heartburn?				
		No		Yes	
		300	43.4%	391	56.6%
		Frequency	%	Frequency	%
Do you suffer from any diseases?	No	262	37.9%	260	37.6%
	Hypertension	5	0.7%	27	3.9%
	Diabetes	10	1.4%	36	5.2%
	Heart diseases	6	0.9%	10	1.4%
	Dyslipidemia	3	0.4%	28	4.1%
	Stroke	0	0.0%	3	0.4%
	Other diseases	14	2.0%	27	3.9%
Have you ever heard about heart attacks?	No	23	3.3%	36	5.2%
	Yes	277	40.1%	355	51.4%
Do you know anyone who has had a heart attack before?	No	125	18.1%	122	17.7%
	Yes	175	25.3%	269	38.9%
Have you ever received any information related to heart attacks?	No	99	14.3%	115	16.8%
	Yes				39.8%
Does sudden heart attack require prompt treatment?	No	25	3.6%	32	4.6%
	Yes	285	39.8%	359	52.0%
	Take them to hospital.	143	20.7%	170	24.6%
If someone shows signs and symptoms of a heart attack, what do you think should do first?	Call his/her doctors	9	1.3%	20	2.9%
	Call an ambulance	146	21.1%	196	28.4%
	Contact their family	2	0.3%	5	0.7%
	Other actions	0	0.0%	0	0.0%
If you want to call an ambulance, do you know the phone number?	No	60	8.7%	60	8.7%
	Yes	240	34.7	331	47.9%
Have you heard about the risk factors of heart attacks?	No	76	11.0%	84	12.2%
	Yes	224	32.4%	307	44.4%

**Table 4:** Level of awareness

	Questions	Correct Answer	Percentage of Respondents with the Correct Answer
1	Have you ever heard about heart attacks?	Yes	91.5%
2	Do you know anyone who has had a heart attack before?	Yes	64.3%
3	Have you ever received any information related to heart attacks?	Yes	68.9%
4	Does sudden heart attack require prompt treatment?	Yes	91.8%
5	If someone shows signs and symptoms of a heart attack, what do you think you should do first?	Call an ambulance	49.5%
6	If you want to call an ambulance, do you know the phone number?	Yes	82.6%
7	Have you heard about the risk factors of heart attacks?	Yes	79.8%
	Mean		75.5%

**Table 5:** Association between diagnosis with GERD or heartburn and other factor

Pearson Chi-Square Tests		
Variables	Have you ever been diagnosed with GERD or heartburn?	
Do you suffer from any diseases?	Chi-square	46.942
	Df	6
	Sig.	.000*
Have you ever heard about heart attacks?	Chi-square	0.516
	Df	1
	Sig.	.004*
Have you ever received any information related to heart attacks?	Chi-square	0.88
	Df	1
	Sig.	0.348
Does sudden heart attack require prompt treatment?	Chi-square	0.005
	Df	1
	Sig.	0.944
If someone shows signs and symptoms of a heart attack, what do you think should do first?	Chi-square	3.168
	Df	3
	Sig.	.366
If you want to call an ambulance, do you know the phone number?	Chi-square	2.563
	Df	1
	Sig.	0.234

**Table 6:** Sources of information

Source	N	Percent
TV	153	13.90
Internet	308	28.10
Books	173	15.80
Media	149	13.60
Promotional leaflets	31	2.80
Health care professionals	230	21.00
Seminars	53	4.80

**Table 7:** Risk factors for a heart attack

Risk	N	Percent
Smoking	549	13.90
Obesity	571	14.50
Diabetes	331	8.40
Exercise	34	0.90
Unhealthy diet	314	8.00
Stress	358	9.10
Alcohol	240	6.10
Genetic	219	5.50
Atrial Fibrilation	183	4.60
High Cholesterol	386	9.80
Lack of Exercise	286	7.20
Heart Diseases	457	11.60
I Don’t Know	20	0.50

**Table 8:** Symptoms of a heart attack

Symptoms	N	Percent
Sudden pain or discomfort in jaw, neck, or back	280	15.20
Weakness or dizziness	207	11.20
Sudden pain or discomfort in the chest	482	26.20
Sudden disturbance of vision in one or both eyes	170	9.20
Sudden pain or discomfort in the arms or shoulders	311	16.90
Sudden shortness of breath	391	21.20

## Discussion

The current study assessed the awareness of early signs and symptoms of AMI among GERD and non-GERD patients in Saudi Arabia and their knowledge of the best course of action to be taken from the onset of symptoms. The result showed that 43.6% of participants were male and 56.4% were female. Out of the respondents, 56.6% had received a diagnosis of GERD, and 43.3% had not received such a diagnosis. The result demonstrated a high level of awareness toward AMI in both groups of respondents (GERD and non-GERD), which was higher than expected. Moreover, there was a significant difference in the level of awareness according to an educational level only. Conversely, there were no significant differences between males and females and between GERD/non-GERD diagnoses. The knowledge of proper action in response to AMI was low. The result showed a high level of knowledge of AMI in GERD patients. This might be due to the disease itself; it could encourage patients to be more aware of complications and other diseases that share similar risk factors. The present findings seem to be consistent with other research, which found high levels of knowledge of AMI (60.4%) among hypertensive patients [15]. Similarly, a study conducted in Nepal illustrated that more than half of the participants knew that AMI could be a complication of diabetes mellitus [16]. Additionally, there were no significant differences between participants who had GERD and those who did not have GERD regarding knowledge of AMI. This might be explained by the fact that most participants had a high level of education. The most common symptoms identified by the participants were sudden pain in the chest, followed by a sudden shortness of breath and sudden pain in the arm or shoulder. However, a study in the United States reported that the most common symptoms of AMI are chest pain and jaw, neck, or back pain. Furthermore, the knowledge of the most common symptoms of AMI was higher in that study compared to this one [17]. The most prevalent risk factors recognized among respondents were smoking (13.90%) and obesity (14.50%). This aligns with a study that was conducted in the western region of Saudi Arabia, where 66.7% of respondents identified smoking as a risk factor [18]. Moreover, two other studies in Saudi Arabia and Kuwait reported very high awareness levels of smoking as a risk factor, reaching over 95% of respondents [19,20]. The results showed that the level of knowledge regarding the appropriate action to be taken in the presence of AMI symptoms (49.5%) was low compared with Korea (67.0%) [21], Poland (87.4%) [22], and the United States (86.8%) [17].

Limitations

This study’s strength was its high enrollment of participants who had been diagnosed with GERD or heartburn; this strengthened the result. However, there were some limitations in the study. First, the database did not contain data on the source of participants’ GERD diagnoses and whether they had been made by a doctor or by the participants themselves. Second, Saudi residents in Saudi Arabia were included in the study, but residents of other nationalities and Saudis residing abroad were not included. Third, the majority of the participants were of young age; the mean age was 31.6, with a standard deviation of 13.66. If there was a greater diversity in respondent age, the study might have shown additional information. Future studies should take these limitations into consideration.

Recommendation

Based on the findings of this study, annual awareness and education campaigns would be appropriate, with a focus on GERD patients and high-risk people, in order to raise knowledge of AMI symptoms and the appropriate course of action that should be taken if symptoms develop.

## Conclusions

The study showed that the overall knowledge and awareness of AMI were suboptimal. The majority of participants had previously heard about AMI, but they were unsure regarding the proper first step to be taken when witnessing signs or symptoms. There was no significant difference in awareness levels among people who reported GERD diagnoses and those who did not. The authors suggest launching additional awareness campaigns about AMI and what to do next and educating people about cardiovascular disease in Saudi Arabia, especially among members of high-risk groups. More studies are needed to identify awareness of GERD complications and also to detect awareness regarding AMI among other patients.
